# Measuring emergency department crowding in an inner city hospital in The Netherlands

**DOI:** 10.1186/1865-1380-6-21

**Published:** 2013-07-08

**Authors:** Martijn Anneveld, Christien van der Linden, Diana Grootendorst, Martha Galli-Leslie

**Affiliations:** 1 Medical Centre Haaglanden, Postbox 432, 2501, CK, The Hague, The Netherlands; 2 Landsteiner Institute, Medical Centre Haaglanden, Postbox 432 2501, CK The Hague, The Netherlands

**Keywords:** Emergency department, Crowding, NEDOCS, National emergency department overcrowding study

## Abstract

**Background:**

Overcrowding in the emergency department (ED) is an increasing problem worldwide. In The Netherlands overcrowding is not a major issue, although some urban hospitals struggle with increased throughput. In 2004, Weiss et al. created the NEDOCS tool (National Emergency Department Over Crowding Study), a web-based instrument to measure objective overcrowding with scores between 0 (not busy at all) to above 181 (disaster). In this study we tried to validate the accuracy of the NEDOCS tool by comparing this with the subjective feelings of the ED nurse and emergency physician (EP) in an inner city hospital in The Netherlands.

**Methods:**

In a 4-week period, data of a total of 147 time samplings were collected. The subjective feelings of being overcrowded and feeling rushed by the ED nurse and EP were scored on a survey using a 6-point Likert scale on answering the question of how busy they would say the ED is right now. NEDOCS tool scores were calculated, and these were compared with the subjective feelings using the kappa statistic assessing linear weights according to Cohen’s method.

**Results:**

Of all the time samplings, approximately 80% of the surveys were completed. The ED was rated as overcrowded 9% of the time by the ED nurses and 11% of the time by the EPs. The median NEDOCS score was 37 (0 to 120) and scored as overcrowded in 3%. There was a good intrarater agreement for the ED nurse and EP for the feeling of overcrowding and feeling of being rushed (κ = 0.79 and 0.73, respectively); the interrater agreement was moderate (κ = 0.53 and 0.43, respectively). The agreement between the NEDOCS and the subjective variables was moderate (κ = 0.50 and 0.53, respectively). A composite variable was created as the average of both the scores of feeling overcrowded of the nurse and the EP and the score of the EP of feeling rushed. The agreement between this and the NEDOCS was κ = 0.53.

**Conclusions:**

The NEDOCS tool is a reasonably good tool to quantify the subjective feelings of overcrowding. When overcrowding is encountered and immediately recognised, specific measures can be taken to guarantee the timely provision of necessary medical care to the patients in the ED at that time. However, possibly more accurate agreements could be obtained as approximately 20% of the surveys were not completed because of perceived crowdedness. An important limitation is that only 3% of the NEDOCS is scored as overcrowded, so no conclusions can be drawn about the agreement for higher categories of overcrowding. It is suggested to repeat the study in a busier period. As the triage category was not taken into account in the formula, a high workload with only a few patients giving high scores in subjective overcrowding in spite of a low NEDOCS score could have led to lower agreements. Incorporating the triage category in the NEDOCS tool possibly will lead to better agreement, but further research is needed to assess this idea.

## Background

Hospital emergency departments (EDs) are experiencing an increase in patient volume, which results in limitations to access and crowding [1,2]. Factors explaining this increase include changes in demography and rising community expectations regarding ED access [2]. ED crowding has become a public health problem where periodic supply and demand mismatches in the ED and hospital resources cause long waiting times and delay in critical treatment [3]. In The Netherlands, ED crowding is not a major issue [3]. However, some urban hospitals do struggle with increased throughput.

At present, there is no standard definition for overcrowding. Crowding is influenced by the number of patients and medical personnel, the number of beds in the ED and the number of available beds in the hospital as well as by waiting times for laboratory results and radiology examinations and the availability of consulting specialists. Earlier, Weiss et al. [4] developed the NEDOCS (National Emergency Department Over Crowding Study) tool to quantitatively describe the staff’s sense of overcrowding. This is a web-based calculator, which converts a simple data set into a score that correlates accurately with the degree of overcrowding as perceived by the senior staff working at that time [4]. There are other quantitative scales to measure overcrowding in the literature: the Real-time Emergency Analysis of Demand Indicators (READI), the Emergency Department Work Index (EDWIN) and the Emergency Department Crowding Scale (EDCS) [5-7]. Of these four, the NEDOCS showed the best discriminative properties for ED overcrowding as shown by the highest area under the receiver-operating characteristic curve (AROC) [5,6,8], while the EDCS had the poorest discriminative properties for ED overcrowding [6].

The purpose of this study is to validate the accuracy of a crowding instrument (NEDOCS tool) by direct comparison with subjective assessment of crowding by the attending emergency physician (EP) and emergency department nurse. The Medical Centre Haaglanden (MCH) Westeinde hospital is a level 1 trauma centre with 52,000 visits per year at the ED in one of the most densely populated areas of the country. In the region it serves as the primary hospital for neurological emergencies including neurological trauma and vascular neurological emergencies, as well as for large trauma and cardiac emergencies. Furthermore, as it is situated in the centre of The Hague city, there are many self-referred patients (about 60%) who bypass their GP and visit the ED directly for a diversity of major and minor problems. The impression of overcrowding is often perceived in our hospital by the nurses and the attending physicians in the ED, although it has never been measured.

The main outcome measures of this study were:

–  Measurements of the NEDOCS score.

–  The degree of overcrowding as well as the impression of being rushed, as rated by the ED nurse and the EP, using a 6-point Likert scale. Based on previous research [4] a combined outcome variable (composite variable) was created that consisted of the average response of the nurse’s and EP’s opinions of ED overcrowding and feelings of being rushed.

–  The agreement between the scores of the ED nurse and EP.

–  The agreement between the scores of the ED nurse, EP and the composite variable with the NEDOCS score.

## Methods

### Study design

A prospective, observational study was performed between 18 June 2012 and 15 July 2012.

### Study protocol

Collection of ED overcrowding data was performed by the attending EP, ED nurse and researchers. These data were collected at 9 a.m., noon, 3 p.m., 6 p.m., 9 p.m. and midnight. Samplings at 3 a.m. and 6 a.m. were omitted because, on average, these times are relatively slow.

### Subjective overcrowding

The attending EP and the coordinating ED nurse were independently approached to answer the following question [5]:

“How busy would you say the ED is right now? Please take into account your workload, the workload of all attending doctors and nurses in the ED, the number of patients in the ED and waiting room, and numbers of holds (admitted patients waiting for beds)”. This was registered on a Likert scale (1 not busy at all, not crowded; 2 busy; 3 extremely busy but not overcrowded; 4 overcrowded; 5 severely overcrowded; 6 dangerously overcrowded). An even Likert scale was used so that the breakpoint for overcrowded versus not overcrowded fell between 3 and 4 [4]. The degree of “feeling rushed” was registered in the same way for the ED nurse and the attending EP (Appendix A). In accordance with Weiss, a composite variable was created as the average of both the scores of feeling overcrowded of the nurse and the EP and the score of the EP of feeling rushed [4].

### Objective overcrowding

The NEDOCS scores were collected by the researchers and attending physicians and consisted of the following [4]:

1. Total number of patients in the ED occupying beds (including waiting area, hallways, etc.).

2. Total number of patients on ventilators.

3. Total number of patients awaiting admission.

4. Waiting time for the last patient called in from the waiting room.

5. Longest time the patient waits for admission.

6. Number of beds in the ED.

7. Number of total beds (occupied and vacant) in hospital (i.e. the number of beds that could be used in case of a disaster).

These data were entered into the web-based calculator (http://hsc.unm.edu/emermed/nedocs_fin2009.shtm) [9] created by Weiss et al. [4].

The scores were divided into six categories (0–20 not busy; 21–60 busy; 61–100 very busy; 101–140 overcrowded; 141–180 dangerous; >181 disaster).

The study was exempted by the Dutch Medical Ethics Committee. Patient consent was not required.

### Statistical analysis

Statistical analysis was performed with SPSS 17.0 and Vassarstats: Website for Statistical Computation (http://www.vassarstats.net) [10]. Agreement between subjective measures of overcrowding, objective measures of overcrowding and NEDOCS was assessed by weighted kappa statistic (κ). The weights assigned were calculated according to Cohen’s method [11] using linear weights. κ can have a maximum value of 1, indicating perfect agreement.

## Results

During a 4-week period, data from six time samplings on every day were collected. This created a database of 168 samplings with NEDOCS scores and Likert scores on the impression of overcrowding and feelings of being rushed as noted by the nurse and the EP. A total of 3,990 patients visited the ED during the study period, which was an average of 142.5 patients per day (range from 120–171, SD 12.6). In Table 1 the scoring of the objective variables is shown.

**Table 1:** Results of objective scoring

	**Median**	**IQR**
No. patients in ED	14	10 - 20
No. patients waiting for admission	1	0 - 2
Waiting times (h)		
In waiting room	0.1	0 - 0.75
For admission	2.1	0 - 4

Due to maintenance work on our electronic patient tracking system, there were a few moments during which we were not able to register the NEDOCS scores: we omitted these moments, leaving a total of 147 samplings. The nurses completed 130 surveys (88%) and the EPs completed 115 surveys (78%). The NEDOCS was completed 75% of the time.

**Figure 1:**
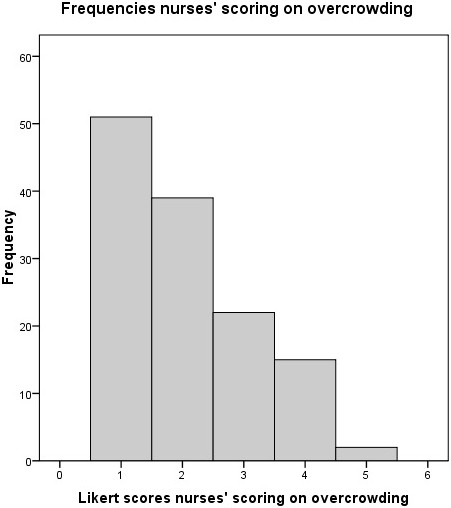
Frequencies nurses’ scoring on overcrowding.

**Figure 2:**
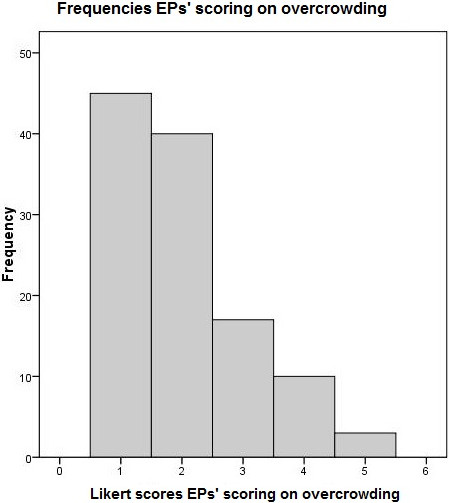
Frequencies EP’s scoring on overcrowding.

The ED nurses rated the ED as overcrowded (>3) in 9% and the EPs in 11% of the samplings (Figures 1 and 2). For the composite variable, overcrowding was calculated in 9% of the cases. Table 2 shows the mean and median scores for the ED nurses’ and EPs’ rates of overcrowding and feeling of being rushed.

**Table 2:** Overall mean subjective ED overcrowding as measured by EP, ED nurse and composite variable

	**Mean**	**SD**
Nurse Overcrowded	2.05	1.09
Nurse rushed	2.02	1.21
EP overcrowded	2.01	1.06
EP rushed	2.27	1.27
Composite variable*	2.13	1.04

*The composite variable for ED overcrowding is the mean of the subjective scores for ED overcrowding of the ED nurse and EP and feeling rushed of the EP.

The weighted Cohen’s kappa agreement for the ED nurses’ and the EPs’ feelings of being overcrowded was κ = 0.53 (95% CI: 0.42-0.64). The weighted kappa for the composite variable with the NEDOCS was κ = 0.53 (95%CI: 0.42-0.63) (Table 3). The intrarater agreement between both the subjective scores for the ED nurse as well as the EP was substantial, κ 0.79 and 0.73 respectively.

**Table 3:** Agreement (weighted Cohen’s kappa) of EPs’ and ED nurses’ feeling rushed and overcrowded with NEDOCS score

		**Nurse**	**EP**	**NEDOCS**
		**Feeling rushed**	**Overcrowded**	
Nurse	Overcrowded	0.79	0.53	0.50
	Feeling rushed	-	-	0.38
EP	Overcrowded	-	-	0.57
	Feeling rushed	0.43	0.73	0.46
	Composite variable*	-	-	0.53

*The composite variable for ED overcrowding is the mean of the subjective scores for ED overcrowding of the ED nurse and EP and feeling rushed of the EP.

To compare the agreement between the subjective scores and the NEDOCS scores, the variables must be in the same range. Therefore we converted the NEDOCS scores to a 6-point scale in accordance with the six categories; hence 0–20 on the NEDOCS would be ranked as 1, 21–60 as 2, and so on. The agreement with the NEDOCS and the feelings of overcrowding was 0.50 and 0.57 for the ED nurse and EP respectively. Table 3 presents the degrees of agreement between the subjective variables and the NEDOCS.

**Figure 3:**
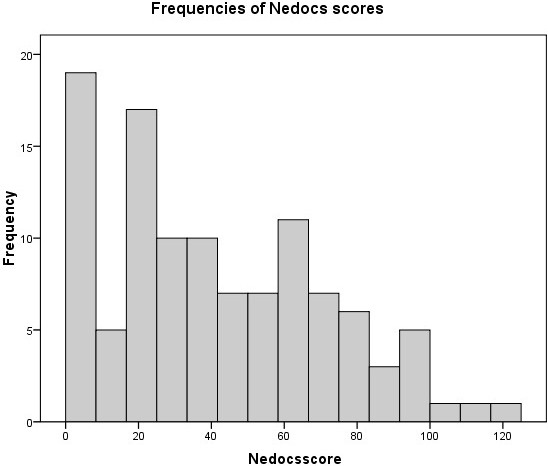
Frequencies of Nedocs score.

The median NEDOCS score was 37 with a minimum of -13 at 9 a.m. and a maximum of 120 at 6 p.m. For practical use we converted scores below 0 to 0, as this has the same clinical implication. Of all the NEDOCS scores, three time samplings scored as overcrowded, which is a score higher than 100 (2.7%) (Figure 3).

## Discussion

Overcrowding in the emergency department is a problem that is often perceived by the staff but hard to determine quantitatively. The circumstances that influence the degree of overcrowding are diverse. Several methods have been developed to measure the degree of overcrowding, including the NEDOCS, the EDWIN and the READI [4,5,7]. Earlier studies showed different results for scores of agreement between the subjective feelings of overcrowding and the objective instruments that measure overcrowding [6,8]. To our knowledge, the NEDOCS has never been validated in The Netherlands. We used this tool to compare the subjective scores of our staff, as this showed the best discriminative properties for overcrowding in the ED [4,6,8]. In this web-based tool, seven variables were needed, resulting in a score between 0 and 200, depicting the degree of overcrowding (0 is not busy and above 181 is a disaster). A number of variables influencing crowding, experienced everyday in the ED, were not taken into account: waiting times for laboratory results, radiology reports and delay in contact with the consulting specialist. Weiss previously described that these variables are difficult to measure accurately, requiring too much data collection and being highly centre-dependent [4]. Furthermore, these variables did not show significance compared to the variables in the NEDOCS tool and were therefore not taken into account in the formula.

In this study we found a good intrarater agreement between the nurses’ feelings of crowding and of being rushed. For the EPs the intrarater agreement was almost as high, which is in accordance with Jones et al. [6]. The agreement between the EP and nurse for both subjective variables was moderate. This could be explained by the fact that the degree of crowding is perceived at different moments for the EP and the ED nurse. When multiple patients are present in the ED, the workload for the ED nurses and EPs will not always be equal at the same moments.

The highest agreements between the subjective and objective methods were those between the NEDOCS and the feelings of overcrowding. For both the ED nurses and the EPs these were fair; between the NEDOCS and the feelings of being rushed these were only moderate. There was a slight tendency to underestimate the NEDOCS with one category, i.e. when the NEDOCS had a score of 2, more often the ED nurses and EPs rated a subjective score of 1 and this led to a lower agreement.

Of all the samplings, the NEDOCS only scored as overcrowded (above 100) three times (2.7%), so the average crowding in this study only ranged from “not busy” to “very busy”. This could be due to the fact that the study was done in a relatively quiet period during the national summer holidays. Although only 2.7% of the NEDOCS scores were 4 or higher, the ED nurses and EPs rated the degree of crowding in 9% and 11%, respectively, as overcrowded. This could imply that with a higher NEDOCS score the subjective feeling is overrated, although these results must be interpreted with caution as such a small proportion of scores was above 100. The difference in scoring with higher scores for the subjective scoring could be due to the fact that a small number of patients may put a high workload on the staff, for example when a major trauma patient and a patient in cardiac arrest are present at the same time in the ED. It seems obvious that the severity of the patients’ conditions influences the degree of overcrowding. To our knowledge, neither of the crowding instruments described earlier takes the triage category into account. The differences between a low NEDOCS score and a high score on the feeling of being rushed could possibly be overcome if each patient is weighed to conform to his or her triage category. In the study setting the Manchester Triage System is used, which classifies the patients into five categories ranging from ‘has to been seen immediately’ to ‘can wait for a maximum of 4 h’. Probably it would be wise to create a kind of patient/severity variable in which the triage category is taken into account instead of counting only the number of patients so the workload with the total number of patients would be more realistic. Further research is needed to assess this idea.

With these tools demonstrating moderate to good agreement, it is possible to quantify the subjective feelings of overcrowding and being rushed. When the staff experiences a feeling of being rushed, the NEDOCS tool can be completed to see if there really is a situation of overcrowding. Of course, some variables must be taken into account that the NEDOCS tool does not provide; as mentioned before, there could be a small number of patients in the ED creating a massive workload for the medical personnel. But if the NEDOCS tool shows a high score then the recommendation could be to implement a protocol to streamline the overcrowding. For instance, the attending EP will critically screen the electronic patient tracking system, evaluate which patients are still in need of care in the ED and which patients could be admitted, discharged or referred for care elsewhere. As a result the degree of overcrowding could decrease and the quality of care remain high. Other options to reduce the volume include diverting ambulances for a limited time or deploying extra medical staff.

Another clinical use of the NEDOCS score could be as an electronic status bar in the electronic patient tracking system that continuously displays the NEDOCS score at a central vantage point. With an up-to-date status of the degree of crowding, actions needed to improve overcrowding can be initiated.

This study has some limitations. First, we experienced some technical difficulty during the maintenance periods of our electronic patient tracking system and therefore had to omit some data. In the remaining 147 time samplings there are still some missing data. Sometimes it was simply forgotten, and other times personnel were too busy to fill in the questionnaires and the NEDOCS tool. It would be expected that some of the missing data would be in the higher range, although this cannot be confirmed. If more data had been available, probably stronger agreements would have been found. To overcome this problem, the help of a research assistant is recommended. Second, this study was done in a rather quiet period of the year, the start of the national summer holidays. In spite of this, there was good agreement with the NEDOCS score and the subjective feelings of experiencing overcrowding. Unfortunately, the score of 3 (very busy) was only reached in 2.7% of the cases on the NEDOCS. Therefore, we cannot determine the agreement in situations of overcrowding (i.e. a score >100). This also explains why the composite variable is lower than calculated by Weiss (mean of 2.13 with an SD of 1.0 vs. a mean of 2.7 with an SD of 1.4) [4]. Repetition of this study in a busier period is suggested to determine the agreement in higher NEDOCS scores and higher scores on the Likert scale. Third, we chose to use Cohen’s kappa to measure agreement, which is suitable for ordinal data. A symmetrical table is required to calculate Cohen’s kappa. The NEDOCS score ranges from 0 to 200 and to compare these variables with the 6-point Likert scale we had to convert the NEDOCS to a 6-point scale in accordance with the six categories; hence 0–20 on the NEDOCS would be ranked as 1, 20–40 as 2, and so on. However, this would suggest a perfect linear score of the NEDOCS and the accuracy of the scores by categorising these into six categories could be doubtful this way. To maximise the rate of reliability we suggest using a VAS-like scale ranging from 0 to 100 and then multiplying by 2 or a VAS scale from 0 to 200 to compare the NEDOCS with the subjective variables.

## Conclusion

The NEDOCS tool is a reasonably good tool to quantify the subjective impressions of overcrowding and being rushed as experienced by ED nurses and attending emergency physicians in the emergency department. When overcrowding is encountered and immediately recognised, specific measures can be taken to guarantee the timely provision of necessary medical care to the patients in the ED at that time.

## Appendix A

### Survey form for the ED physician and ED nurse for the NEDOCS tool

Date:

Please circle the time.

Time: 9 am 12 am 3 pm 6 pm 9 pm 12 pm

Please circle your opinion on “Degree of Overcrowding”

1 2 3 4 5 6

1. Not busy at all, not crowded

2. Busy

3. Extremely busy but not overcrowded

4. Overcrowded

5. Severely overcrowded

6. Dangerously overcrowded.”

Please circle your opinion on “Feeling rushed” in the ED

1 2 3 4 5 6

1= Not rushed

6= Rushed

## Abbreviations

ED: Emergency department; EP: Emergency physician; NEDOCS: National Emergency Department Over Crowding Study.

## Competing interests

The authors declare that there are no competing interests.

## Authors’ contributions

MA performed most of the research, the statistical analysis and wrote this manuscript. CL also performed the research and provided useful contributions to the research and manuscript. DG contributed to the statistical analysis. MG provided valuable comments on the manuscript. All authors read and approved the final manuscript.
